# Polypropylene/Poly(butylene adipate-co-terephthalate) Breathing Film for Inhibiting *Pseudomonas* and Maintaining Microbial Communities and Postharvest Quality of *Allium mongolicum* Regel during Storage

**DOI:** 10.3390/foods12183370

**Published:** 2023-09-08

**Authors:** Hongyu Bu, Jian Hu, Feng Han, Limei Wang, Qianru Chen, Peifang Cheng, Hai Yue, Tungalag Dong, Xueyan Yun

**Affiliations:** 1 College of Food Science and Engineering, Inner Mongolia Agricultural University, Hohhot 010018, China; bhongyu163@163.com (H.B.); hujian0475@163.com (J.H.); qrchen_nmg@163.com (Q.C.); pfcheng2007@163.com (P.C.); dongtlg@163.com (T.D.); 2 Inner Mongolia Institute for Drug Control, Hohhot 010020, China; astronaut0206@sina.com (F.H.); linxiaomin261211@163.com (H.Y.); 3 Inner Mongolia Academy of Agricultural & Animal Husbandry Sciences, Hohhot 010031, China; w524041348@163.com

**Keywords:** polypropylene/poly(butylene adipate-co-terephthalate) breathing film, modified atmosphere packaging, *Allium mongolicum* Regel, *Pseudomonas*, microbial diversity, postharvest quality

## Abstract

*Allium mongolicum* Regel (*A. mongolicum*) is a healthy edible plant but highly perishable with a short shelf life of 1–2 d. Modified atmosphere packaging (MAP) could inhibit the postharvest senescence and decay of the vegetables. Thus, the aim of this study was to apply MAP with different gas permeabilities to the storage of *A. mongolicum* and evaluate its effects on maintaining microbial communities and the postharvest quality of *A. mongolicum*. The results showed that polypropylene/poly(butylene adipate-co-terephthalate) (PP/PBAT, abbreviated as PAT) MAP was suitable for the storage of *A. mongolicum* by establishing an optimal atmosphere of 0.5–0.6% O_2_ and 6.2–7.1% CO_2_ in the bag. It could delay the postharvest senescence of *A. mongolicum* and maintain its quality by slowing down its respiration rate and weight loss, reducing cell membrane permeability and lipid peroxidation, maintaining the cell wall, and reducing infection and the growth of microorganisms. However, *A. mongolicum* in HPT was more perishable than that in PAT during storage. *Pseudomonas* was found to be the main spoilage bacteria, and they could also be effectively inhibited by PAT-MAP. The next-generation sequencing results also showed the growth of *Escherichia-Shigella*, *Clostridium sensu stricto 1*, *Streptococcus*, *Aureobasidium*, *Didymella*, and *Fusarium*, responsible for *A. mongolicum* decay or human disease, was well inhibited by PAT-MAP. The results suggested that PAT-MAP could be used to maintain microbial diversity and the postharvest quality of *A. mongolicum* under cold storage conditions. It provided a feasible solution for the preservation, food quality, and safety control of *A. mongolicum*.

## 1. Introduction

*Allium mongolicum* Regel, Liliaceae, Allium, a bulb-tufted perennial herb, is widely distributed in northwest China [1]. It has high nutritional value, delicious taste, and rich bioactive ingredients. However, *A. mongolicum* is highly perishable after harvest and has a short shelf life of only 1–2 d under room temperature, which poses a significant challenge to the distribution and retail of *A. mongolicum* [2]. Currently, studies on *A. mongolicum* are focused on its nutritional composition and bioactive components [3,4], and minimal information is available regarding its postharvest physiology and quality control.

The postharvest quality loss of fresh fruit and vegetables is mainly caused by mechanical damage, physiological corruption, and microbial attack [5,6,7]. Microbial spoilage has led to the wastage of around 45% of harvested fruit and vegetables worldwide. More than 90% of microbial spoilage is caused by *Pseudomonas* and *Erwinia* [8]. Fresh fruit and vegetables are also frequently contaminated with human gastrointestinal pathogenic bacteria such as *Salmonella*, *Escherichia coli O157:H7*, and *Shigella* [9], which continues to be a significant source of foodborne illness outbreaks. Moreover, the microbial diversity of fresh vegetables is closely related to their quality and safety during storage. It was reported that the microbial diversity of fresh vegetables dropped, and *Pseudomonas* became the predominant bacteria involved in the quality deterioration of fresh fruit and vegetables [10,11]. 

Generally, the postharvest quality loss in fresh fruit and vegetables is controlled by many physical, chemical, and biological treatments, such as cold storage [5], ozone [12], melatonin [13], and antimicrobial coating treatments [6,7], etc. However, most of these have high costs or toxic residues, which are difficult to popularize. Among them, modified atmosphere packaging (MAP), a widely used, economical, and safe approach, could inhibit the postharvest senescence of fruit and vegetables [14,15,16]. MAP with more than 5% CO_2_ could significantly reduce respiration [17], bacterial growth [2,18], lipid peroxidation, and damage to mitochondrial structure [15]. Furthermore, the headspace gas compositions greatly depend on the gas permeability of the packaging film, metabolism characteristics of the product, ambient temperature, and humidity [2]. The permeability properties of most polymeric films commonly used in MAP have limitations. Nonetheless, polypropylene (PP) film, with its low price, high safety, and excellent mechanical capacity, is commonly used for food packaging. However, its poor CO_2_ and H_2_O vapor permeability could cause dew condensation in the bag due to the anaerobic respiration of vegetables and degradation of phenolic compounds, making it not suitable for the packaging of vegetables with a strong respiration metabolism [19,20,21]. However, poly (butylene adipate)-co-terephthalate (PBAT) has high H_2_O vapor and CO_2_ transmission rates, which makes it ideal for improving the permeability of PP film. Furthermore, PBAT is biodegradable and highly compatible, and it has been used to blend with various polymers to enhance permeability [22,23,24]. In the study reported here, PP and PBAT co-extruded to produce a PP/PBAT blend film with moderate gas permeability, matching the storage of *A. mongolicum*. In addition, the degradation performance of PP was improved in the blend film [24].

However, under storage, the postharvest quality and microbial composition of *A. mongolicum,* and the relationship between them, are unknown. Thus, it will be very interesting to carry out this work. In this study, the effects of the MAPs on the postharvest physicochemical and physiological properties and microbial diversity of *A. mongolicum* stored in cold storage were researched. In addition, the relationship between quality deterioration and microbial diversity was revealed. This work contributes to the application of MAP and provides new strategies for the preservation and quality control of *A. mongolicum*.

## 2. Materials and Methods

### 2.1. Materials Preparation and Sample Storage

**Figure 1:**
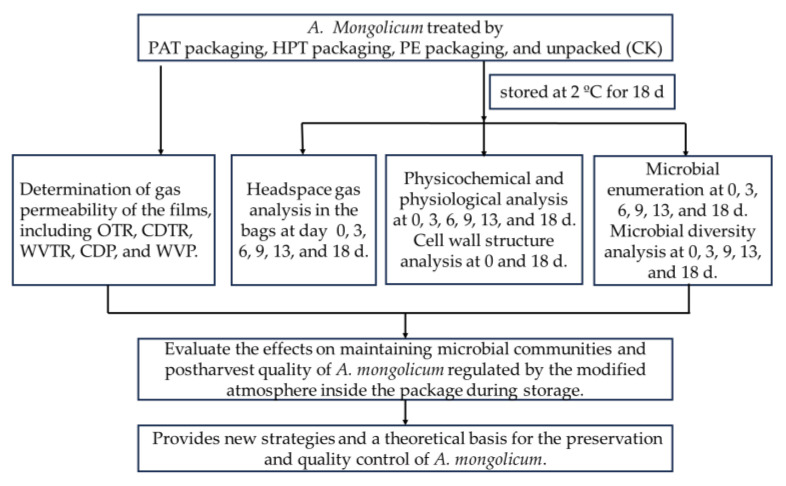
The flow diagram of the experimentation.

The samples with intact leaves, uniform size, and no pests or diseases were taken from the Alxa League farm in China and sent to the laboratory immediately after harvest via cold chain. The new blend film of polypropylene/poly(butylene adipate-co-terephthalate) (PP/PBAT, abbreviated as PAT) was prepared in the laboratory. PBAT (Mn = 1.7 × 10^5^) was purchased from Xinfu Science and Technology Co., Ltd. (Hangzhou, China). PP (Mn = 1.3 × 10^6^) was purchased from Esun Advanced Materials Co., Ltd. (Shenzhen, China). PP/PBAT blend films, a mixture of 70% PP and 30% PBAT, were prepared by a twin-screw extruder system (PPT-3/SJ2-20-250, Guangzhou POTOP Experimental Analysis Instrument Co., Ltd., Guangzhou, China). The temperature of the screw area in the charging barrel was 95–200 °C, and the extrusion temperature of the die head was 200 °C. Then, the blend films were made into 15 cm × 27 cm bags with a heat-sealing machine (DBF-900, Wenzhou Dingli Package Machine Manufacture Co., Ltd., Wenzhou, China). The film thickness was 35 ± 5 μm. Samples (90 ± 2 g) were randomly packed. Four treatments were set up in the experiment. The samples packed with PP/PBAT films were abbreviated as PAT. The samples were soaked in 50 mg L^−1^ non-electrolytic slightly acidic hypochlorous water with pH 6.4 for 5 min, and then rinsed with sterilized distilled water, dried, and packed with PP/PBAT films, abbreviated as HPT. The samples were packed with commonly used PE preservation films (Miaojie, Top Daily Chemicals Co., Ltd., Wuxi, China), abbreviated as PE. The unpacked *A. mongolicum* samples were used as a control, abbreviated as CK. Then, those samples were divided into four groups and stored for 18 d at 2 °C and 75 ± 5% relative humidity. The samples in the four groups were collected at 0, 3, 6, 9, 13, and 18 d during storage, respectively. Three bags of parallel samples in each group were randomly taken for investigation. The diagram (Figure 1) showed the experimentation flow and the analysis performed at each stage.

### 2.2. Gas Permeability of the Film

The gas permeability of the film was evaluated by the carbon dioxide transmission rate (CDTR), oxygen transmission rate (OTR), and water vapor transmission rate (WVTR). The CDTR, OTR, and WVTR were analyzed following the method reported previously [6]. The manometric gas permeability tester (Lyssy L100-5000, Systech Illinois Instruments, Inc., Oxford, UK) was used to test CDTR and OTR at 23 °C. The water vapor permeability meter (Permatran-W Model 3/61, Mocon Inc., Brooklyn Park, MN, USA) was used to test WVTR at a temperature of 23 °C and humidity of 65%. Then, carbon dioxide permeability (CDP) and water vapor permeability (WVP) were calculated.

### 2.3. Headspace Gas Analysis

The contents of O_2_ and CO_2_ in bags were tested using the headspace gas analyzer (Model 6600, Systech Instruments Co., Oxford, UK) according to the method of Bu et al. [2] Gas in the bags was collected and detected with a sampling needle. The results were output in %.

### 2.4. Physicochemical and Physiological Analysis

#### 2.4.1. Texture Profile Analysis

The texture of *A. mongolicum*, expressed by maximum shearing forces, was tested with the texture analyzer (TA-XT Plus, SMS Co., Surrey, UK), following the method outlined previously [25]. The HDP/BSW shearing probe, simulating incisors biting and cutting food, was used to cut off and test the texture of the sample. *A. mongolicum* with both ends fixed on the stage was cut off 6 cm away from the root, and maximum shearing forces were measured. Compression was performed once (distant 15.00 mm, Trigger mode Button) at a rate of 1 mm s^−1^.

#### 2.4.2. Weight Loss and Decay Rate

The initial weight of *A. mongolicum* samples in each bag was weighed by the precision electronic balance (PRECISA JA-5003B, Precisa Co., Gottingen, Germany) based on the method reported previously [12]. The samples were then taken and weighed after storage. The weight loss rate was the percentage of lost weight compared to initial weight, which was expressed as %. The decay rate was the ratio of the rotten sample number to the total sample number in each bag. The results were expressed as %. Rotten samples refer to any occurrence of decay, regardless of the degree of decay.

#### 2.4.3. Respiration Rate

The respiration rate of the samples was tested according to the method used in previous studies [2,12]. The samples (60 ± 5 g) were placed in 1 L sealed tanks for 1 h at 25 °C. Gas composition in the tanks was collected with a sampling needle and measured with a headspace O_2_/CO_2_ analyzer (Model 6600, Systech Instruments Co., Oxford, UK). The results of respiration rate were expressed as mg kg^−1^ h^−1^.

#### 2.4.4. Chlorophyll Content, Lipid Peroxidation, and Membrane Permeability

The chlorophyll content was analyzed according to the method in previous studies [26]. The results of chlorophyll content were calculated as g kg^−1^. The malondialdehyde (MDA) content, indicating the lipid peroxidation level, was tested with thiobarbituric acid following the method outlined previously [27]. The results of MDA content were calculated as μmol kg^−1^. The relative electrolyte leakage conductivity (EC), indicating the membrane permeability, was analyzed based on the method outlined previously [28]. The *A. mongolicum* sample was cut into uniform tissue slices (3 mm diameter, 3 mm thickness), then 5 g sample slices were soaked in 50 mL distilled water for 1 h to wash and remove any surface contamination. Then the sample was taken out and placed into another 50 mL of distilled water, incubated for 3 h at 20 ± 2 °C, and the EC (EC_1_) was tested. The same samples were then put into a water bath at 100 °C for 30 min, then the solution was cooled to 20 °C and the EC (EC_2_) was tested. The results were the percentage of EC_1_ to EC_2_, which was expressed as %.

### 2.5. Morphology of Cell Wall

Cell wall integrity of *A. mongolicum* was analyzed using a scanning electron microscopy (SEM) (TM400, Hitachi Co., Tokyo, Japan) following the method reported previously [29]. The cell wall morphology of *A. mongolicum* was analyzed at 0 and 18 d of storage. The samples were sliced into uniform tissue slices (3 mm diameter, 1 mm thickness) and fixed with 2.5% glutaraldehyde. Then the samples were dehydrated with ethanol according to the gradient concentrations of 30%, 50%, 70%, 80%, 90%, and 100%. Then the *A. mongolicum* samples were dried with a vacuum freeze drier and observed with the SEM at a 15 kV accelerating voltage and high-vacuum mode after being coating with gold.

### 2.6. Microbial Enumeration

The samples (25 ± 0.1 g) were weighed and added to 225 mL of buffered peptone water, then the solution was shaken for 30 min to prepare 10^−1^ dilute solution. Then series diluents of 10^−2^, 10^−3^, 10^−4^, 10^−5^, 10^−6^, and 10^−7^ were prepared using a 10-fold dilution method. Aerobic bacterial counts (ABC) were analyzed based on the method outlined previously [30]. Psychrophile plate counts (PPC) were tested using the method outlined previously [31]. *Pseudomonas* counts (PC) were analyzed using the method reported previously [11]. Yeast and mold counts (YAM) were determined following the method outlined previously [2]. The results of colonies’ numbers were all expressed as Log_10_ CFU g^−1^.

### 2.7. Microbial Profile Analysis

#### 2.7.1. High-Throughput Illumina Sequencing

A CTAB method was used to extract the total genome DNA of *A. mongolicum* samples. After testing the purity and concentration of DNA, an appropriate amount of the sample was put into a centrifuge tube, and the sample was diluted to 1 ng μL^−1^ with sterile water. PCR reactions are performed using diluted genomic DNA of 10 ng as a template, 2 μmol L^−1^ of forward and reverse primers with the barcode according to distinct sequencing region (16S V4: 515F-806R, ITS1: ITS5-1737F, and ITS2-2043R), and 15 μL of Phusion^®^ High-Fidelity PCR Master Mix with GC Buffer from New England Biolabs to ensure amplification efficiency and accuracy. PCR products were tested using 2% agarose gel electrophoresis, and then recovered for the target strips using the Gel Extraction Kit provided by Qiagen of Germany. TruSeq^®^ DNA PCR-Free Sample Preparation Kit from Illumina in the USA was used to build the sequencing library. After the constructed library was qualified by the detection of Qubit@ 2.0 Fluorometer from Thermo Scientific and Bioanalyzer 2100 system from Agilent, libraries were sequenced using NovaSeq6000 (Illumina, San Diego, CA, USA).

#### 2.7.2. Data Analysis

FLASH (Version 1.2.7) was used to merge the paired-end reads, and the QIIME quality-controlled process (Version 1.9.1) was used to filtrate data of the raw tags. Then the effective tags were obtained after removing the chimera sequences with UCHIME. Effective tags in all samples were clustered using Uparse software (Version 7.0.1001), and sequences with 97% consistency were assigned to the same OTUs. The Silva and Unite databases were used for 16S and ITS species annotation [32,33], respectively. The biomarker OTUs, differing in abundance and occurrence between samples, were analyzed with the linear discriminant analysis (LDA) effect size (LEfSe) algorithm. The significance level was set at LDA ≥ 4 and *p* < 0.05. The Ade4 package and the ggplot2 package in R software (Version 2.15.3) were used to perform Principal Coordinate Analysis (PCoA).

### 2.8. Statistical Analysis

Statistical differences were calculated using Duncan’s multiple range test using the SPSS software (version 26.0) System. The significance level was set at *p* < 0.05. The results were presented as mean ± standard deviation.

## 3. Results and Discussion

### 3.1. Gas Permeability of the Film

The ideal gas atmosphere in the package could extend the shelf life of fruit and vegetables, and the gas content in the package mainly depends on the gas permeation performance of the packaging materials and the characteristics of the packaging products and their interaction [25]. The gas permeation performance of materials is commonly represented by their transmission rate and permeability; permeabilities such as CDP and WVP are essential properties of materials and are not related to their thickness [6]. As shown in Table 1, the CDP and WVP of PP were significantly lower than that of PBAT. The poor CDP and WVP of PP could cause dew condensation in the bag and anaerobic respiration of vegetables and are not suitable for the packaging of vegetables with a strong respiration metabolism. PBAT is biodegradable and highly compatible [23,24] but has high CDP and WVP, which are also not suitable for the packaging of vegetables. As shown in Table 1, the blend of PP and PBAT, abbreviated as PAT, had moderate CDP and WVP, matching the storage of *A. mongolicum*. The blending improved the permeation performance of both PP and PBAT. This is because the PBAT components in an amorphous structure are mainly dispersed in the continuous phase of PP, and the internal free volume of PBAT is large, providing a channel for the transmission of CO_2_ and H_2_O. Furthermore, it was found that the CDP and WVP of the commonly used PE preservation film were significantly lower than those of PAT and even lower than PP. Thus, the internal atmosphere of high CO_2_ and low O_2_ could be formed in the PAT bags to inhibit the respiration rate of *A. mongolicum*. However, there were few studies on the recommended CO_2_ and O_2_ levels for *A. mongolicum* storage. However, for vegetables, the concentrations of O_2_ from 0.0% to 5.0% and CO_2_ greater than 5% were the recommended gas concentration for their storage [17,34].

### 3.2. Headspace Gas Analysis

Suitable gas composition was conducive to maintaining the quality of fresh agricultural products after harvest and prolonging their shelf life [12,30]. As shown in Table 2, the results showed that the O_2_ concentration decreased sharply within 3 d of storage, and then tended to be stable, fluctuating between 0.51 and 0.63% and 0.05 and 0.09% in PAT and HPT treatment, respectively. For PE packages, it fluctuated between 19.53 and 20.33%. The CO_2_ concentration in PAT and HPT packages rapidly reached peak values of 8.03% and 10.73% within 3 d, and then tended to be stable at 6.20–7.07% and 6.40–9.20%, respectively (Table 2). It should be noted that the O_2_ level was higher and the CO_2_ level was lower in PAT compared to HPT during the entire storage, indicating PAT-treated samples have a lower respiration rate, less O_2_ consumption, and less CO_2_ generation, while HPT-treated samples have a higher respiration rate [30]. Moreover, it was also reported that the concentration of O_2_ from 0.0% to 5.0% was the best gas concentration for vegetable storage [34], and 6.13% CO_2_ concentration was conducive to maintaining the postharvest quality of green vegetables [35]. In addition, combined with the analysis data of the physicochemical properties and microbial structure of *A. mongolicum* in different groups, it was found that PAT-MAP could better match the respiration of *A. mongolicum* and could form an ideal gas composition with 0.5–0.6% O_2_ and 6.2–7.1% CO_2_ suitable for *A. mongolicum* storage.

### 3.3. Physicochemical Analysis

#### 3.3.1. Maximum Shearing Forces

The tenderness of *A. mongolicum* was expressed through its maximum shearing forces. The maximum shearing forces in all treatments increased and then decreased during storage (Table 3). The maximum shearing forces in PAT remained relatively steady, fluctuating from 2.84 N to 3.29 N, which maintained a better texture and tenderness of the sample than other treatments. The maximum shearing forces decreased sharply after day 6 of storage in PE and HPT, which was caused by the softening and decay of tissues.

#### 3.3.2. Weight Loss and Decay Rate

The weight loss rate is an essential indicator for evaluating the postharvest quality of fruit and vegetables. When it is greater than 5%, they lose market value [36]. During storage, the weight loss rate in each treatment increased (Table 3). However, it increased slowly in PAT and was only 4.64% during the entire storage. The weight loss rate in HPT and PE were more significant than 5% on day 9 of storage and rose rapidly during the late storage period. The decay rate is another essential indicator of the storage effect of fruit and vegetables. They will lose market value when the decay rate exceeds 10%. As shown in Table 3, the decay rate in each treatment increased during storage. However, the decay rate in PAT increased slowly and was only 4.95% by the end of storage. The decay rates in HPT and PE were 23.28% and 30.78%, respectively, which were 4.7 and 6.2 times that in PAT. The rapid decay in PE was mainly due to the vigorous respiration of *A. mongolicum* and serious dewing in the PE package. Dewing may have caused the rapid propagation of microorganisms that infected the tissue and caused tissue softening and corruption.

#### 3.3.3. Respiration Rate

**Figure 2:**
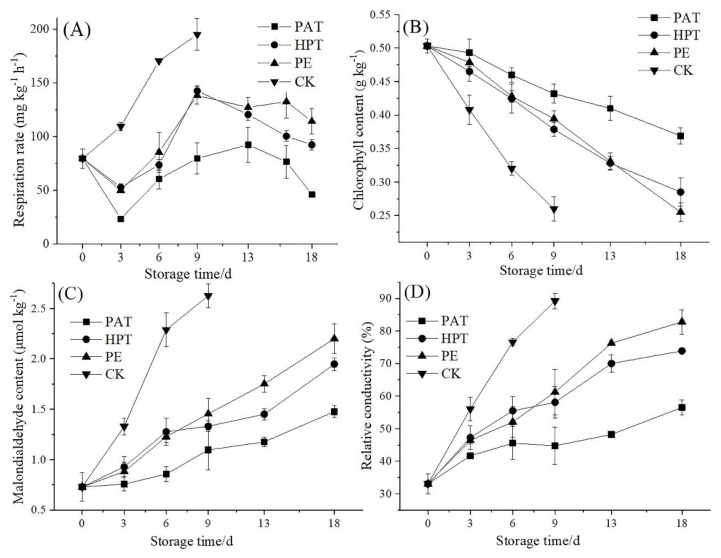
Effects of different treatments on the respiration rate (**A**); chlorophyll content (**B**), malondialdehyde content (**C**), and relative conductivity (**D**) of *A. mongolicum* stored at 2 °C for 18 d. The data expressed the mean of 3 determinations. PAT: *A. mongolicum* packaged with PP/PBAT blend film, HPT: *A. mongolicum* washed in non-electrolytic slightly acidic hypochlorous water and then packaged with PP/PBAT blend film, PE: *A. mongolicum* packaged with polyethylene film. CK: *A. mongolicum* unpackaged.

Respiration is the primary physiological metabolism of postharvest *A. mongolicum*, directly affecting the shelf life and quality of *A. mongolicum*. As shown in Figure 2A, during entire storage, the respiration rate in PAT, HPT, and PE were significantly lower than CK (*p* < 0.05). The respiration rate in PAT was the lowest (*p* < 0.05). For the PAT, HPT, and PE groups, the respiration rate decreased within 3 d, then increased and reached its peak on the 9th day in HPT and PE and on the 13th day in PAT, followed by a decline. The respiration peak values of *A. mongolicum* in HPT, PE, and PAT bags were 142.64 mg kg^−1^ h^−1^, 138.27 mg kg^−1^ h^−1^, and 92.43 mg kg^−1^ h^−1^, respectively. These results indicated that PAT treatment significantly reduced the respiration rate of *A. mongolicum* and delayed the appearance of its respiration peak.

#### 3.3.4. Total Chlorophyll Content

A degradation of the total chlorophyll content indicates the postharvest vegetable quality loss associated with cellular degradation and senescence [37]. The chlorophyll content in each treatment showed a decreasing trend with increasing storage time (Figure 2B). However, in PAT, it fell slowly, reaching 0.37 g kg^−1^ by the end of storage. The total chlorophyll content in HPT and PE was 0.29 g kg^−1^ and 0.26 g kg^−1^, respectively, which were 21.6% and 29.7% lower than that in PAT. This may be due to the PAT treatment inhibiting the chlorophyll-degrading enzyme’s activity, thereby reducing the degradation of chlorophyll [38]. Furthermore, the oxidative decomposition of chlorophyll can be directly caused by the generation of ROS [15]. Therefore, it could be concluded that PAT could inhibit the degradation of chlorophyll by reducing ROS. Furthermore, PE treatment with high O_2_ and low CO_2_ partial pressure could induce chlorophyll degradation [39].

#### 3.3.5. Membrane Permeability and Lipid Peroxidation

Membrane lipid peroxidation and electrolyte leakage were caused by ROS accumulation in the cell after harvest [40]. Membrane lipid peroxidation was assessed via malondialdehyde (MDA), and membrane permeability was evaluated via relative electrolyte leakage conductivity (EC). As shown in Figure 2C,D, the MDA and EC increased in all treatments during storage. However, the MDA and EC in PAT increased slowly compared to other groups (*p* < 0.05). They were 1.48 µmol kg^−1^ and 56.50% at the end of storage, respectively. The MDA and EC in PE were 2.20 µmol kg^−1^ and 82.78%, respectively, which were 48.6% and 46.4% higher than those in PAT. The higher lipid peroxidation level in PE may be caused by high O_2_ and low CO_2_ in the package [39]. The MDA and EC in HPT were 1.95 µmol kg^−1^ and 73.86% at the end of storage, which were 31.8% and 73.86% higher than those in PAT. These results revealed that the elevated CO_2_ concentration of 6.2–7.1% and the low O_2_ concentration of 0.5–0.6% in the PAT bags might decrease the superoxide anion and hydroxyl radical [15], thereby delaying membrane damage and lipid peroxidation. It was also reported that the disrupted ROS metabolic balance resulted in damage to the cell membrane system and the postharvest senescence of vegetables [41].

### 3.4. Morphology of Cell Wall

**Figure 3:**
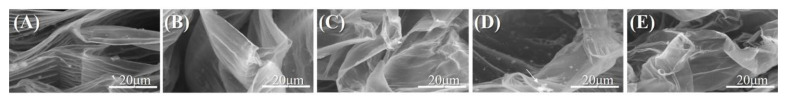
Microstructure (**A**–**E**) of *A. mongolicum* tissue in different treatments during storage observed with SEM at 15 kV accelerating voltage and high-vacuum mode after coating with gold. (**A**) Fresh sample (2000×); (**B**) PAT at 18 d (2000×); (**C**) HPT at 18 d (2000×); (**D**) PE at 18 d (2000×); (**E**) CK at 18 d (2000×).

The integrity of the cell wall structure is also an important indicator of the storage effect of *A. mongolicum*. As shown in Figure 3A, the cell wall structures in fresh *A. mongolicum* samples were intact. At the end of storage, the PAT-treated cell wall structure was more intact with a smooth cell wall edge compared with other treatments (Figure 3B). However, the cell wall was ruptured in HPT (Figure 3C) and CK (Figure 3E). The cell wall microstructure in PE was also broken, manifesting as blistering and wrinkling of the cell wall (Figure 3D). The results indicated that PAT-MAP could form a suitable atmosphere for *A. mongolicum* preservation, which could protect the integrity of the cell wall in *A. mongolicum* samples, thereby maintaining the quality and delaying the senescence of *A. mongolicum*. It was also reported that MAP maintained banana fruit quality under storage by improving the antioxidant system and cell wall structure [42]. It is worth noting that microbes invaded the *A. mongolicum* samples in PE more severely than other treatments (arrowhead in figures), as illustrated in Figure 3D. Moreover, microbes entered the tissues and tore the network of the cell wall, thus mechanically destroying the cell wall [29].

### 3.5. Microbial Enumeration Analysis

**Figure 4:**
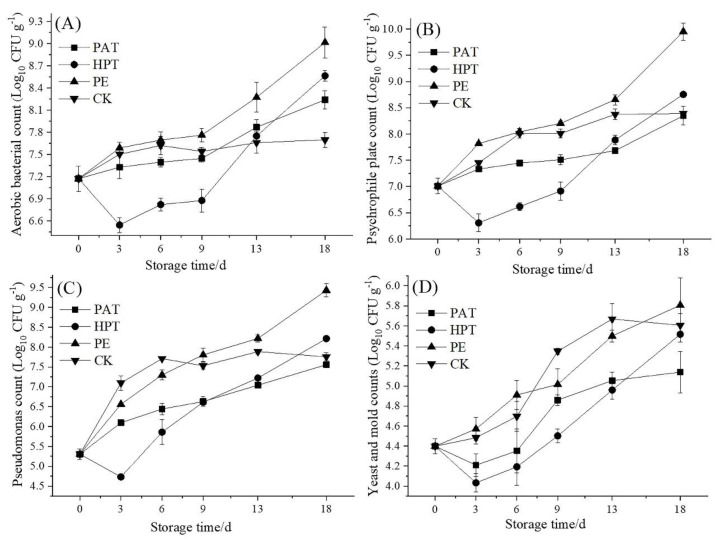
Effects of different treatments on the aerobic bacterial count (**A**), psychrophile plate count (**B**), *Pseudomonas* count (**C**), yeast and mold counts (**D**) of *A. mongolicum* stored at 2 °C for 18 d. The data expressed the mean of 3 determinations.

The postharvest quality and safety improvement in vegetables rely on a better understanding of microbial population dynamic changes during storage. As shown in Figure 4A–C, it was found that the ABC, PPC, and PC were increased in all treatments except HPT during storage. HPT was found to be more effective in reducing the initial microbial load but then rebounded after day 3 (*p* < 0.05), which is consistent with the research results reported by Xiao et al. (2014) [26]. The rebound may be caused by residual spore germination or the recovery of damaged bacteria after sterilization during the following storage [43].

The ABC, PPC, and PC in PE increased quickly and maintained a higher level than those in PAT and HPT during storage, which reached 9.01 Log_10_ CFU g^−1^, 9.95 Log_10_ CFU g^−1^, and 9.43 Log_10_ CFU g^−1^ at the end of storage. Moreover, the YAM in PE increased rapidly and reached 5.81 Log_10_ CFU g^−1^ at the end of storage (Figure 4D). It was also found that the microbial population of vegetables maintained significantly high levels in PE packages during storage [40]. The higher level of microbial load may be caused by the high O_2_ level, low CO_2_ level, and the dew in PE packages, which could boost bacterial growth and then result in decay of *A. mongolicum* (Table 2 and Table 3). However, the ABC, PPC, PC, and YAM in PAT increased much more slowly and were 0.77 Log_10_ CFU g^−1^, 1.60 Log_10_ CFU g^−1^, 1.87 Log_10_ CFU g^−1^, and 0.67 Log_10_ CFU g^−1^ lower than PE at the end of storage. Thereby, PAT-MAP could inhibit microbial growth and maintain excellent quality due to its higher CO_2_ content of 6.2–7.1%, lower O_2_ content of 0.5–0.6%, and minimum water loss of *A. mongolicum* in PAT packages (Table 2 and Table 3).

Moreover, at the end of storage, the ABC, PPC, and YAM in all treatments increased by 1.07–1.84 Log_10_ CFU g^−1^, 1.34–2.94 Log_10_ CFU g^−1^, and 0.74–1.41 Log_10_ CFU g^−1^, respectively, while the PC increased by up to 2.26–4.13 Log_10_ CFU g^−1^. The results revealed that *Pseudomonas* increased fastest and were the dominant bacteria during the storage of *A. mongolicum*. In addition, the amounts of *Pseudomonas* in PE were highest and reached 9.43 Log_10_ CFU g^−1^. The highest level of decay rate was also found in PE. Thus, based on the above data, it could be inferred that *Pseudomonas* was the main spoilage bacteria in the *A. mongolicum* samples. *Pseudomonas* was also found to be the dominant bacteria causing the rot of *Agaricus bisporu* [11].

### 3.6. Microbial Profile

#### 3.6.1. Bacterial Profile

**Figure 5:**
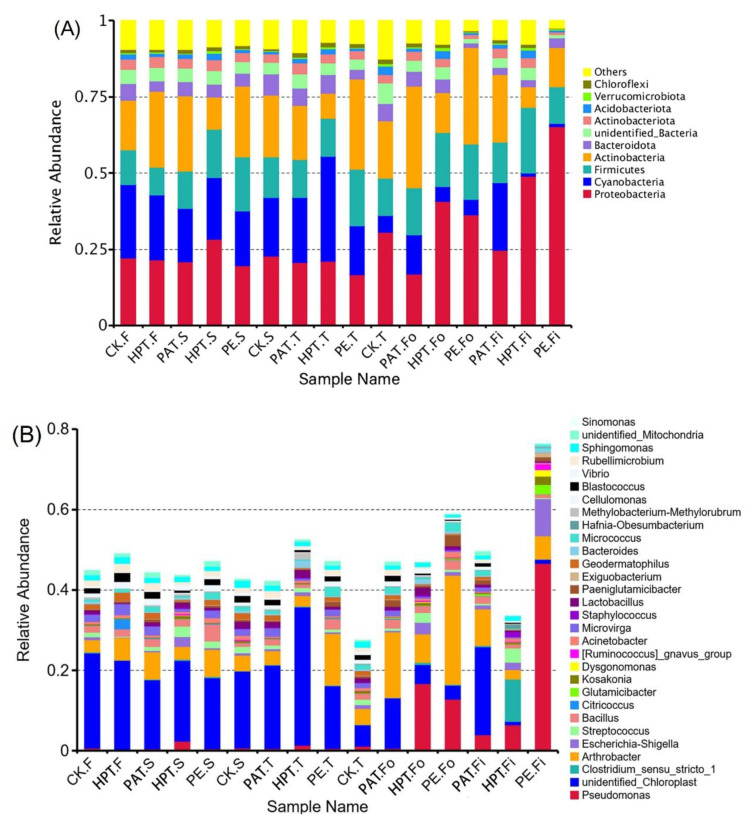

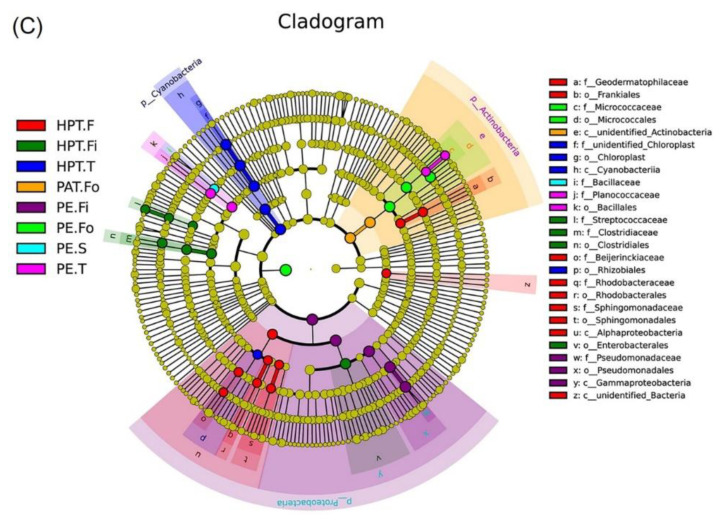

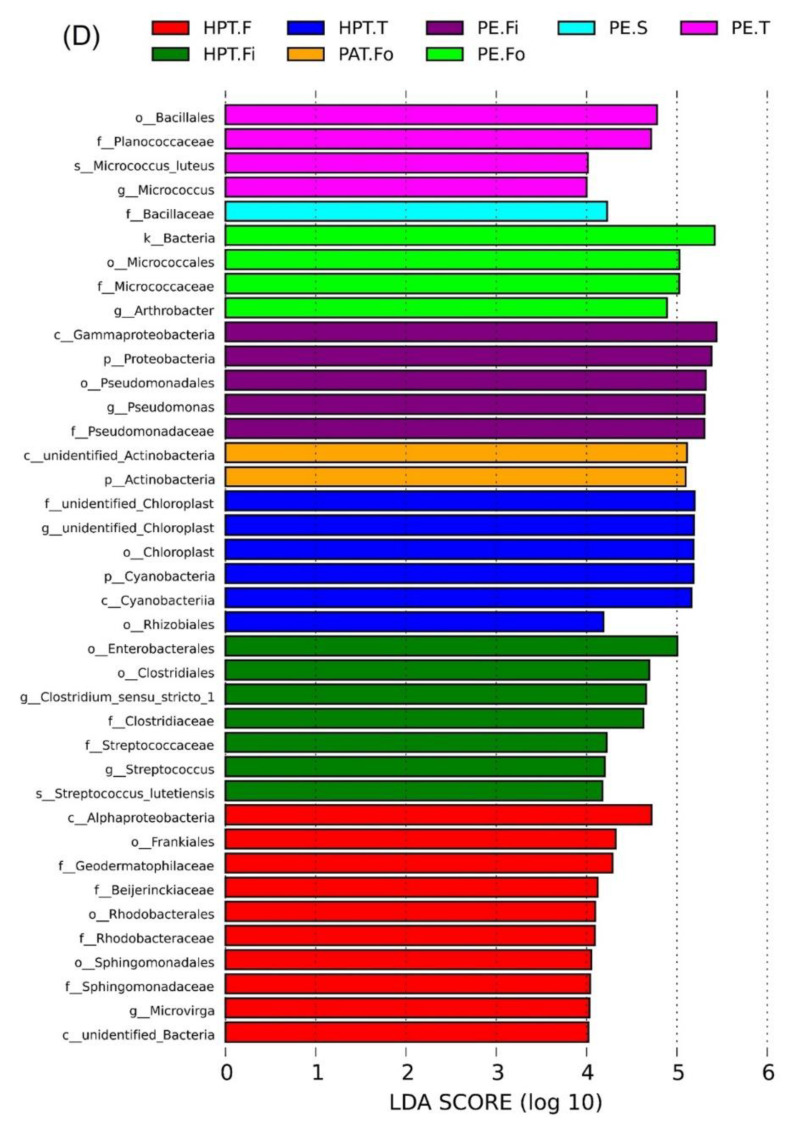

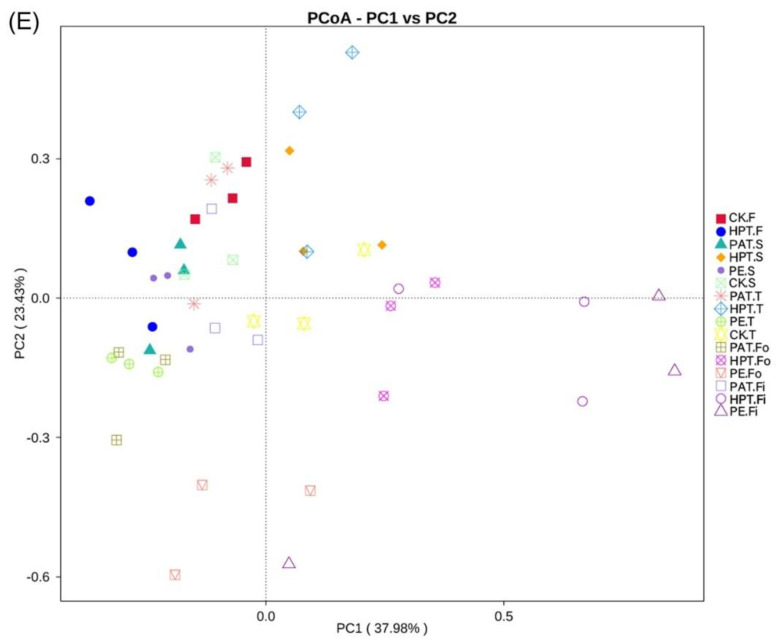
Relative abundance of bacteria communities in *A. mongolicum* samples at phylum (**A**) and genus levels (**B**); bacterial cladogram (**C**); bacteria LDA value distribution histogram (**D**); and PCoA analysis (**E**) in *A. mongolicum* samples. The suffix F of the sample name indicates the first sampling on day 0 of storage; S means the second sampling on day 3 of storage; T marks the third sampling on day 9 of storage; Fo indicates the fourth sampling on day 13 of storage; Fi means the fifth sampling on day 18 of storage. All data represent the mean of 3 determinations.

The taxonomic levels, consisting of phylum, class, order, family, genus, and species, were used to distinguish the microbial community structure [44]. For the bacterial communities, Proteobacteria, Cyanobacteria, Firmicutes, Actinobacteria, and Bacteroidota were the dominant bacteria during storage at the phylum level, accounting for more than 73% of all OTUs in each sample (Figure 5A). Notably, the relative abundance of Proteobacteria increased significantly from 0.22% to 0.65% in PE, and from 0.21% to 0.49% in HPT. Proteobacteria are a major phylum of bacteria that include many pathogens, such as *Escherichia*, *Salmonella*, *Helicobacter*, etc.; some are also plant pathogens [12]. However, Proteobacteria were significantly inhibited in PAT and only increased by 0.03%.

At the genus level (Figure 5B), the relative abundance of *Pseudomonas* in PE increased rapidly from 0.07% to 46.6% during storage. The highest decay rate was also found in PE (Table 3). In addition, *Pseudomonas* accounted for 16.7% of the HPT.Fo samples. Severe decay was also found in the HPT.Fo samples. However, *Pseudomonas* in the PAT bags were inhibited. The best quality and lowest decay rate of *A. mongolicum* were also found in PAT. These results further indicated that the spoilage of *A. mongolicum* could be mainly caused by *Pseudomonas*. *Pseudomonas* were also found to be the main bacteria resulting in the decay and flavor change of wild morel mushrooms [45]. *Pseudomonas fluorescens* was the primary microorganism responsible for the spoilage of rose flowers [46]. *Pseudomonas* was widely distributed in nature; many of them were associated with human infection, such as *P. aeruginosa*, *P. pseudomallei*, and *P. fluorescens*, and many of them caused vegetable decay [47]. These reports further supported the above conclusions of this study. In addition, it was noteworthy that *Clostridium sensu stricto 1* in HPT increased at later storage due to anaerobic conditions with O_2_ below 0.1%. Furthermore, the relative abundance of the foodborne pathogen *Escherichia-Shigella* in PE increased and accounted for 9.2% at the end of storage. It was in accordance with the result that Enterobacteriaceae and Streptococcaceae were the predominant bacteria at the later storage of fresh-cut Ma bamboo [48].

For an in-depth understanding of the difference in the bacterial communities of *A. mongolicum* in different treatments, an LEfSe analysis was conducted (Figure 5C,D). As shown in Figure 5C, 39 bacterial clades were found with LDA scores equal to or greater than 4.0, indicating that these were classified as the most important bacteria to distinguish *A. mongolicum* samples. The 39 clades consist of 1 kingdom, 3 phyla, 5 classes, 10 orders, 11 families, 7 genera, and 2 species. As shown in Figure 5D, the genus *Pseudomonas* was the primary marker for PE at the end of storage. At the same time, *Clostridium sensu stricto 1* and *Streptococcus*, which are pathogenic to humans, were the primary markers for HPT. However, PAT could inhibit the growth of *Pseudomonas*, *Escherichia-Shigella*, *Clostridium sensu stricto 1*, and *Streptococcus*.

To gain an overall understanding of the microbial variations in *A. mongolicum* caused by different treatments, a PCoA analysis was performed. As shown in Figure 5E, the first two extracted components explained more than 61.4% of the bacteria variance in *A. mongolicum* during storage. The samples in PAT clustered together during storage, while the samples were far separated in CK, PE, and HAT. The PCoA of all the samples suggested that PAT-MAP could effectively control and maintain the microbial composition and quality of *A. mongolicum* during storage.

#### 3.6.2. Fungal Profile

**Figure 6:**
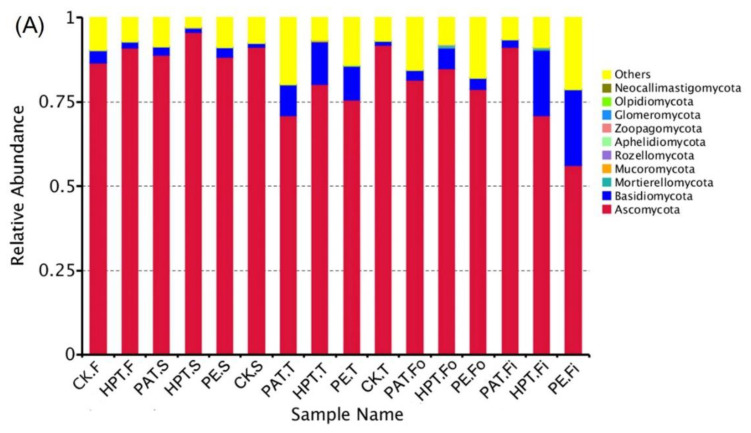

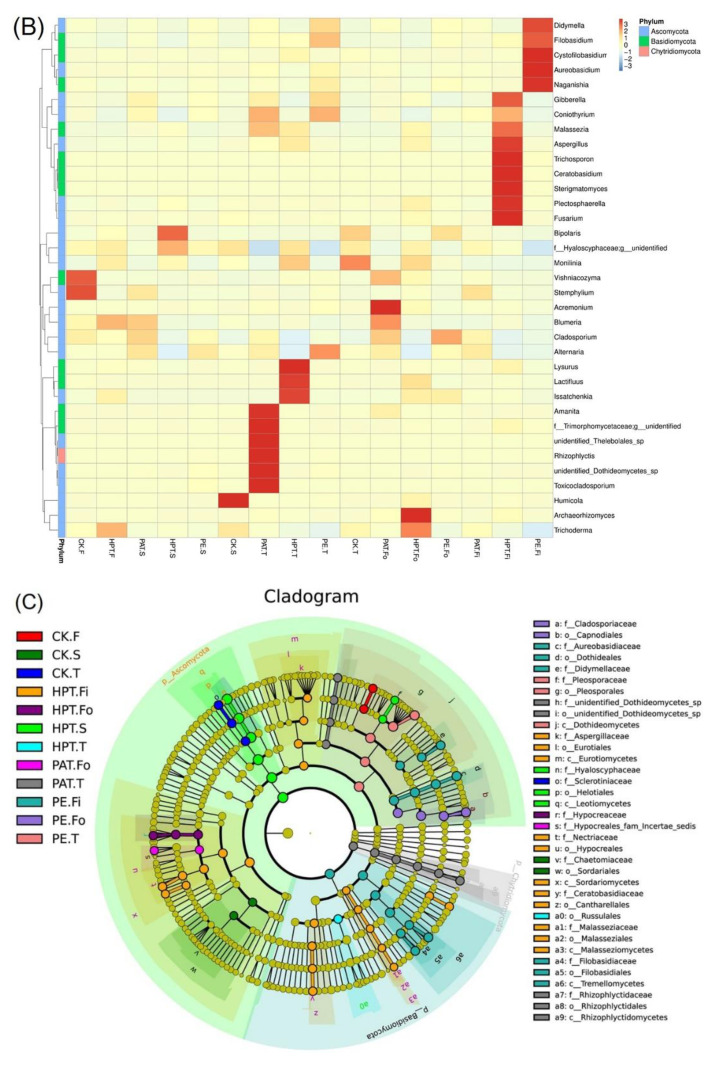

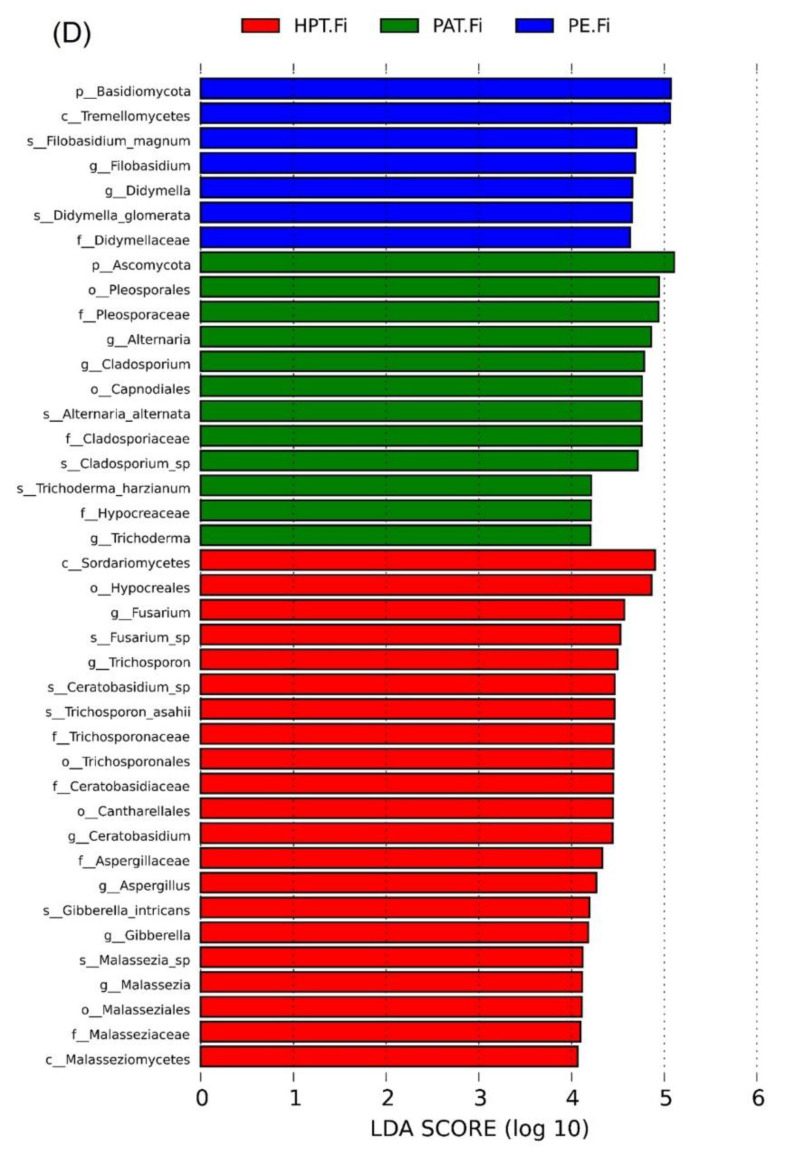

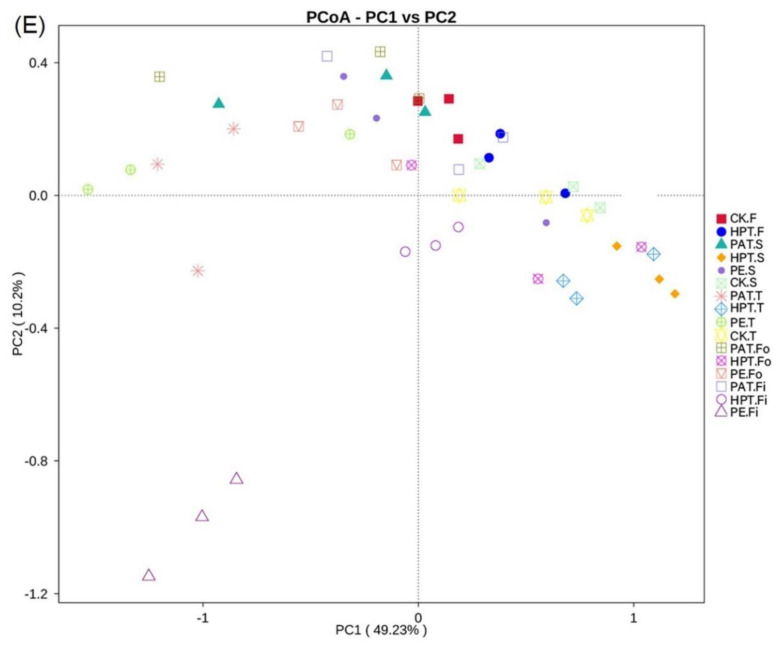
Relative abundance of fungal communities in *A. mongolicum* samples at phylum (**A**); heatmap of fungal communities at the genus level (**B**); fungal Cladogram (**C**); fungal LDA value distribution histogram (**D**); and PCoA analysis (**E**) in *A. mongolicum* samples. The suffix F of the sample name indicates the first sampling on day 0 of storage; S means the second sampling on day 3 of storage; T marks the third sampling on day 9 of storage; Fo indicates the fourth sampling on day 13 of storage; Fi means the fifth sampling on day 18 of storage. All data represent the mean of 3 determinations.

For the fungal communities, over 78% of the sequence reads were annotated as Ascomycota and Basidiomycota at the phylum level in each sample (Figure 6A). Regarding the genus level (Figure 6B), the relative abundance of *Vishniacozyma* and *Stemphylium* in fresh samples decreased during storage. The relative abundance of *Aureobasidium*, *Naganishia*, *Filobasidium,* and *Didymella* in PE increased rapidly and accounted for 25.2%, 10.5%, 9.8%, and 7.8% in all OTUs at the end of storage, and *Filobasidium* could cause vegetable decay [40]. Furthermore, *Fusarium*, *Trichosporon*, *Ceratobasidium*, *Aspergillus*, and *Gibberella* in HPT accounted for more than 3% of all OTUs.

To further understand the difference in fungal composition of *A. mongolicum* in different treatments, an LEfSe analysis was conducted (Figure 6C,D). As shown in Figure 6C, 77 fungal clades were found with LDA scores equal to or greater than 4.0, indicating that these were classified as the most important fungal species to distinguish *A. mongolicum* samples. The 77 clades consist of 3 phyla, 7 classes, 13 orders, 16 families, 19 genera, and 19 species. As shown in Figure 6D, at the end of storage, *Filobasidium* and *Didymella* were the primary markers for PE. Among them, some *Didymella* species were fungal plant pathogens. Moreover, *Fusarium*, *Trichosporon*, *Ceratobasidium*, *Aspergillus*, *Gibberella,* and *Malassezia* were the major markers for HPT. Among them, some Fusarium species are the most important fungal pathogens to plants and humans. Furthermore, Allium species are susceptible to Fusarium. Many Fusarium species could cause Allium species plant rot, such as *F. solani* [49], *F. proliferatum* [50], and *F. falciforme* [51], etc. However, PAT treatment could inhibit the growth of the fungal species pathogenic to plants and humans, which would maintain the quality and guarantee the food safety of *A. mongolicum*.

To gain an overall understanding of the fungal variations in *A. mongolicum* caused by different treatments, a PCoA analysis was performed. As illustrated in Figure 6E, the first two extracted components explained more than 59.4% of the variance in the fungal profile. The samples were gradually separated into CK, PE, and HPT during storage. Especially in PE, the samples shifted from the top-right side to the bottom-left side during storage. While the samples in PAT were still closely clustered during storage. The results suggested that PAT-MAP could effectively inhibit fungal variations and maintain the fungal composition of A. mongolicum during storage.

## 4. Conclusions

A PAT film with an optimal CDTR, OTR, and WVTR could form a suitable gas composition of O_2_, CO_2,_ and H_2_O in the packaging for *A. mongolicum* storage. It could effectively delay postharvest senescence and maintain the quality of *A. mongolicum*. Compared to the PE package commonly used as a preservative film, the respiration rate, decay rate, malondialdehyde, and relative electrolyte leakage conductivity in PAT decreased by 59.6%, 83.9%, 32.7%, and 31.7%, respectively. In addition, the aerobic bacterial counts, psychrophile plate counts, *Pseudomonas* counts, and yeast and mold counts decreased by 0.77 Log_10_ CFU g^−1^, 1.60 Log_10_ CFU g^−1^, 1.87 Log_10_ CFU g^−1^, and 0.67 Log_10_ CFU g^−1^. Furthermore, it could effectively inhibit *Pseudomonas*, *Escherichia-Shigella*, *Clostridium sensu stricto 1*, and *Streptococcus*, which are bacterial pathogens to plants and humans, and *Aureobasidium*, *Didymella*, and *Fusarium*, which are fungi pathogenic to plants and humans. The PAT-MAP extended the shelf life of *A. magnolicum* to 18 days, which is 9 days longer than that of universal PE packaging. Therefore, the application of PAT packaging could provide a feasible solution for preventing postharvest senescence, maintaining quality, and prolonging the shelf life of *A. mongolicum*. This research work provides new strategies and a theoretical basis for the preservation and quality control of *A. mongolicum.*

## Figures and Tables

**Table 1:** The CO_2_, O_2_, and H_2_O vapor permeation performance of the films.

Sample	Thickness (μm)	OTR (cm^3^ m^−2^ d^−1^)	CDTR (cm^3^ m^−2^ d^−1^)	CDP (10^−6^ cm^3^ m m^−2^ d^−1^ Pa^−1^)	WVTR (g m^−2^ d^−1^)	WVP (10^−8^ g m m^−2^ d^−1^ Pa^−1^)
PP	25.0	4119 ± 35	12,451 ± 468	3.08 ± 0.12	2.45 ± 0.30	2.97 ± 0.36
PBAT	25.1	4059 ± 60	38,333 ± 586	9.53 ± 0.15	27.49 ± 0.95	33.5 ± 1.16
PAT	34.2	2990 ± 115	13,867 ± 29	4.70 ± 0.01	4.42 ± 0.15	7.34 ± 0.25
PE	8.0	35,663 ± 182	152,500 ± 534	1.21 ± 0.02	13.77 ± 0.75	0.54 ± 0.05

Results were expressed as mean ± standard deviation, n = 6. CDTR, OTR, and WVTR were carbon dioxide transmission rate, oxygen transmission rate, and water vapor transmission rate. CDP and WVP were carbon dioxide permeability and water vapor permeability. PP: polypropylene film, PBAT: poly(butylene adipate-co-terephthalate) film, PAT: PP/PBAT blend film, PE: polyethylene film.

**Table 2:** Changes in O_2_ and CO_2_ content in packaging bags during storage.

Treatment	Storage Time (d)					
	0	3	6	9	13	18
O_2_ (%)						
PAT	20.5	0.47 ± 0.01 ^b^	0.57 ± 0.10 ^b^	0.54 ± 0.08 ^b^	0.63 ± 0.05 ^b^	0.51 ± 0.01 ^b^
HPT	20.5	0.07 ± 0.01 ^c^	0.06 ± 0.02 ^c^	0.09 ± 0.02 ^c^	0.07 ± 0.01 ^c^	0.05 ± 0.01 ^c^
PE	20.5	19.63 ± 0.21 ^a^	19.87 ± 0.21 ^a^	20.33 ± 0.12 ^a^	19.83 ± 0.12 ^a^	19.53 ± 0.29 ^a^
CO_2_ (%)						
PAT	0	8.03 ± 0.40 ^b^	7.07 ± 0.38 ^b^	6.27 ± 0.25 ^b^	6.53 ± 0.25 ^b^	6.20 ± 0.20 ^a^
HPT	0	10.73 ± 0.68 ^a^	8.77 ± 0.32 ^a^	9.20 ± 1.27 ^a^	7.35 ± 0.78 ^a^	6.40 ± 0.79 ^a^
PE	0	0.37 ± 0.09 ^c^	0.20 ± 0.10 ^c^	0.23 ± 0.06 ^c^	0.23 ± 0.06 ^c^	0.27 ± 0.06 ^b^

Results were expressed as mean ± standard deviation, n = 3. Different superscript letters within a column were significantly different (*p* < 0.05). PAT: *A. mongolicum* packaged with PP/PBAT blend film, HPT: *A. mongolicum* washed in non-electrolytic slightly acidic hypochlorous water and then packaged with PP/PBAT blend film, PE: *A. mongolicum* packaged with polyethylene film.

**Table 3:** Effects of different treatments on the maximum shearing forces, weight loss rate, and decay rate of *A. mongolicum* stored at 2 °C for 18 d.

Treatment	Storage Time (d)					
	0	3	6	9	13	18
Maximum shearing forces (N)						
PAT	2.84 ± 0.49	2.97 ± 0.45 ^c^	3.05 ± 0.56 ^c^	3.29 ± 0.24 ^b^	3.28 ± 0.28 ^a^	2.99 ± 0.32 ^a^
HPT	2.84 ± 0.49	3.88 ± 0.55 ^b^	3.94 ± 0.59 ^b^	3.07 ± 0.62 ^bc^	2.74 ± 0.63 ^b^	2.59 ± 0.44 ^b^
PE	2.84 ± 0.49	2.89 ± 0.38 ^c^	3.11 ± 0.53 ^c^	2.95 ± 0.21 ^c^	2.63 ± 0.40 ^b^	2.55 ± 0.35 ^b^
CK	2.84 ± 0.49	4.68 ± 0.82 ^a^	4.55 ± 0.33 ^a^	4.22 ± 0.20 ^a^		
Weight loss rate (%)						
PAT	0	2.53 ± 0.20 ^c^	2.95 ± 0.24 ^c^	3.03 ± 0.22 ^c^	3.56 ± 0.83 ^c^	4.64 ± 0.76 ^c^
HPT	1.64 ± 0.9	3.60 ± 1.07 ^b^	3.81 ± 0.41 ^b^	5.57 ± 0.85 ^b^	6.92 ± 0.92 ^b^	9.89 ± 1.72 ^b^
PE	0	2.46 ± 0.33 ^c^	2.85 ± 0.07 ^c^	5.68 ± 0.90 ^b^	7.17 ± 0.55 ^b^	10.03 ± 1.33 ^b^
CK	0	29.12 ± 3.34 ^a^	54.00 ± 0.57 ^a^	70.37 ± 1.53 ^a^		
decay rate (%)						
PAT	0	0	0	0.96 ± 0.16 ^c^	1.92 ± 0.38 ^c^	4.95 ± 1.02 ^d^
HPT	0	0	2.67 ± 0.40 ^b^	6.39 ± 0.96 ^b^	11.83 ± 2.02 ^b^	23.28 ± 1.21 ^c^
PE	0	0	0	6.39 ± 1.23 ^b^	15.28 ± 2.41 ^b^	30.78 ± 1.92 ^b^
CK	0	12.86 ± 1.18 ^a^	26.33 ± 1.53 ^a^	31.67 ± 2.89 ^a^		

Results were expressed as mean ± standard deviation. The data of maximum shearing forces represent the mean of 30 determinations, and other data expressed the mean of 3 determinations. Different superscript letters within a column were significantly different (*p* < 0.05). PAT: *A. mongolicum* packaged with PP/PBAT blend film, HPT: *A. mongolicum* washed in non-electrolytic slightly acidic hypochlorous water and then packaged with PP/PBAT blend film, PE: *A. mongolicum* packaged with polyethylene film. CK: *A. mongolicum* unpackaged.

## Data Availability

The data used to support the findings of this study can be made available by the corresponding author upon request.
